# Recruitment of adults with moderate eczema for a randomised trial: Comparison of traditional versus modern methods

**DOI:** 10.1111/ajd.13699

**Published:** 2021-09-03

**Authors:** Fabrizio Spada, Ian P. Harrison, Tanya M. Barnes, Kerryn A. Greive, Daisy Daniels, Joshua P. Townley, Niyaz Mostafa, Andrew T. Fong, Philip L. Tong, Stephen Shumack

**Affiliations:** ^1^ Ego Pharmaceuticals Victoria Australia; ^2^ St George Dermatology and Skin Cancer Centre New South Wales Australia; ^3^ Faculty of Medicine University of New South Wales New South Wales Australia; ^4^ The Children’s Hospital at Westmead New South Wales Australia; ^5^ Department of Dermatology St Vincent’s Hospital New South Wales Australia; ^6^ The Skin Hospital New South Wales Australia; ^7^ Department of Dermatology Royal North Shore Hospital New South Wales Australia

**Keywords:** atopic dermatitis, randomised controlled trial, social media, Eczema Area Severity Index, transepidermal water loss, hydration

## Abstract

**Background:**

Clinical trial recruitment is challenging for investigators who often overestimate the pool of qualified, willing subjects. Moreover, there is a paucity of literature, particularly in dermatology, regarding recruitment and the comparative success of advertising strategies.

**Methods:**

Both ‘traditional’ (physician referral, newspaper and radio advertisements, letterbox drops, posters/flyers, word‐of‐mouth) and ‘modern’ (patient recruitment services, social media, Google advertisements, websites, email) recruitment methods were used to enrol 100 patients (>18 years) diagnosed with moderate eczema for a randomised, double‐blind, placebo‐controlled trial investigating the efficacy and safety of a topical eczema treatment over 4 weeks. The relationships between recruitment method and patient age, sex, race, study completion and costs were analysed.

**Results:**

The majority of patients recruited were young, with millennials and Gen Z comprising 77% of the study population. Both traditional and modern recruitment methods were equally successful in recruiting younger patients, with older patients predominately recruited by traditional methods. Eighty per cent more men were recruited by traditional compared to modern methods, whilst 67% more women than men were recruited by modern methods. Recruitment method neither appeared to be influenced by race, nor did it effect whether patients completed the study. Costs per enrolment were similar for both methods.

**Conclusions:**

This study shows that despite the high proportion of young patients and the rising popularity of social media and increased internet use, a combination of both traditional and modern recruitment methods was required to successfully meet the trial enrolment target of 100 adult patients with moderate eczema.

**Abbreviations**
EASI: Eczema Area and Severity Index
RCT: randomised controlled trial

**What this Research Adds**

Clinical trial recruitment is often a difficult and time‐consuming process. While recruiting for a trial of moderate eczema patients, we have found that a combination approach to recruitment can be a viable and successful strategy. Such an approach should incorporate both modern recruitment methods, such as social media, and more traditional methods, such as letter box drops and newspaper ads, to successfully target as many potential candidates as possible.

## INTRODUCTION

The randomised controlled trial (RCT) is widely accepted as the most powerful research method for minimising bias when evaluating the efficacy and safety of healthcare interventions.^1^ Recruitment and retention of protocol‐appropriate subjects are key to the success of RCTs.^2^ However, clinical trial recruitment is challenging for most investigators, particularly those attempting to enrol large numbers of subjects into trials that make great demands on subjects. Investigators frequently overestimate the pool of available subjects who meet the inclusion criteria and are willing to participate in a particular trial by many fold.^3^

A study of 114 multicentre trials in the United Kingdom found that less than a third achieved their original recruitment target, and up to half had to be extended.^4^, ^5^ In a similar study of 41 trials in the USA, only a third met or exceeded their recruitment target, whilst almost a quarter failed to recruit more than half the required subjects.^6^ A more recent study of 2579 trials suggests that 19% of trials may be terminated due to poor recruitment.^7^ Whilst there are several possible consequences of poor recruitment, perhaps the most crucial is the potential for a trial to be underpowered.^8^ In such circumstances, clinically relevant differences may be reported as statistically non‐significant, increasing the chance that an effective intervention will either be abandoned before its true value is established, or delayed as further trials or meta‐analyses are conducted.^9^ Poor recruitment may also have practical, financial and ethical consequences.^10^, ^11^

To meet recruitment targets for RCTs, investigators have traditionally used newspaper, radio or television advertisements, flyer drops into letterboxes and posters pinned on notice boards or displayed on buses. However, increased internet and social media use in recent times has resulted in these platforms being incorporated into recruitment advertising strategies to augment enrolment.^12^ Social media may offer distinct benefits compared with traditional methods due to its ability to target specific populations using customised messages that may resonate better with the target audience.^13^ Furthermore, some social media platforms have a higher proportion of users from minority groups.^13^, ^14^ Research evaluating the effectiveness of social media in enhancing RCT recruitment in various settings has produced conflicting results. Some studies find social media more effective and less costly than traditional methods,^15^, ^16^, ^17^ whilst others do not^18^, ^19^ or are inconclusive.^20^

Despite the challenges to successful recruitment for RCTs, there is limited literature regarding the recruitment process, particularly in the dermatology field, and the comparative success of various advertising strategies is varied. The aim of this study was to describe our experience using both ‘traditional’ and ‘modern’ recruitment methods for a Phase 2 RCT of a topical treatment for moderate eczema in adults.

## METHODS

### Study design

The study was entered in the Australian New Zealand Clinical Trial Registry on 28 July 2015 (registration number: ACTRN12615000782538), and was conducted between September 2015 and October 2019.^21^ Ethics approval was obtained from Bellberry Limited, Eastwood, South Australia, Australia (approval number: HREC2015‐04‐270), and all patients gave their written informed consent prior to participation.

Briefly, a total of 100 patients (>18 years) with moderate eczema were recruited using a variety of both traditional and modern methods by a single centre (St George Dermatology and Skin Cancer Centre, Kogarah, New South Wales, Australia). Potential participants were screened at the clinic and those meeting the inclusion criteria were enrolled in the trial and randomly assigned to either the active or placebo group. A detailed outline of the trial protocol has been previously described.^21^

Study data were queried for recruitment source, which was subsequently categorised as either ‘traditional’ or ‘modern’. Further analyses were conducted to determine the relationships between traditional versus modern recruitment methods and patient age, sex, race and study completion. The costs per enrolment for both recruitment methods were also calculated.

### Recruitment methods

Traditional recruitment methods included as follows: physician referral of existing patients; an A frame poster placed outside the trial site clinic; flyers placed on the local university notice board, in local GP clinics and in local pharmacies; wobblers placed on shelves in local pharmacies; one local newspaper advertisement; two letterbox drops of approximately 90,000 flyers each advertising the study in the local area surrounding the trial site clinic and covering approximately 58,000 properties; two radio advertisements played at breakfast, mid‐morning and afternoon every day for 2 weeks covering the wider Sydney area; and word‐of‐mouth.

Modern recruitment methods included as follows: two patient recruitment services (TrialFacts, Melbourne, Victoria, Australia and TrialSpark, New York, New York, USA), who utilised social media including Facebook and Instagram, websites and emails to recruit patients; social media advertisements utilising Facebook and Instagram, initially targeting 18–24 year olds within a 9 km radius of the trial site clinic, which later expanded to all ages within a 32 km radius; Google advertising in a similar fashion to social media; an advertisement on the clinical trial sponsor's (Ego Pharmaceuticals, Braeside, Victoria, Australia) website as well as on their Facebook page; and an advertisement on the Eczema Association of Australasia website, as well as in their electronic newsletter.

### Statistical analysis

Percentage increase was calculated as follows: Percentage increase = (final value − starting value)/(starting value) × 100.

## RESULTS

Overall, there were 987 expressions of interest recorded over the duration of the study (between September 2015 and October 2019). Recruitment sources leading to each expression of interest were recorded from 25 April 2016, when the relative success of different advertising strategies was noted, as shown in Table 1. Whilst there were more expressions of interest for traditional compared with modern recruitment methods (504 versus 341), both led to a similar proportion of patients enrolled in the trial (49 versus 40).

**Table 1:** Number of expressions of interest and patients enrolled for traditional and modern methods of recruitment over the study period

Recruitment source	Expressions of interest	Patients enrolled
Unknown	142	11
Traditional methods		
Physician referral	41	14
Newspaper	37	5
Radio	157	7
Letterbox	125	5
Posters/flyers/wobblers	18	1
Word‐of‐mouth	126	17
Total traditional methods	504	49
Modern methods		
Patient recruitment services	138	21
Google advertising	40	4
Social media	140	15
Website	11	0
Email	12	0
Total modern methods	341	40

**Figure 1:**
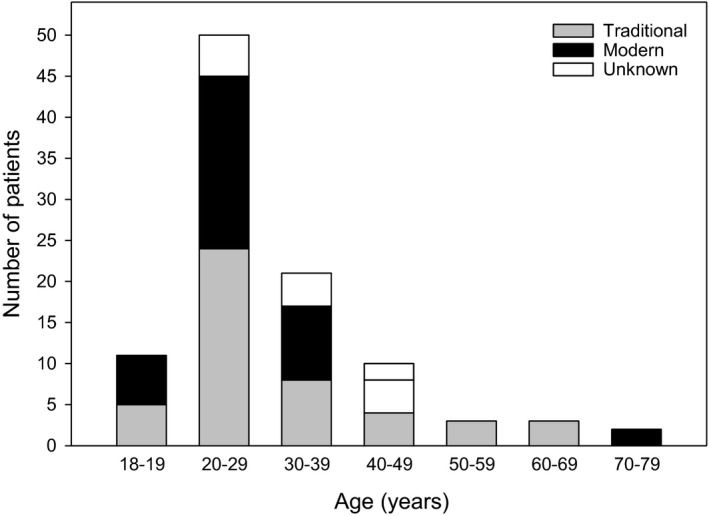
Age distribution of patients categorised by recruitment method.

The mean age of patients enrolled in the trial was 30.9 ± 12.7 years, ranging from 18‐73 years (Figure 1). The overwhelming majority of patients were young Gen Z (13%) or millennials (64%), whilst older Gen X and baby boomers accounted for 15% and 8% of patients, respectively. Patients in the younger age groups were similarly recruited by traditional and modern methods; however, older patients were predominately recruited by traditional methods (Figure 1).

**Figure 2:**
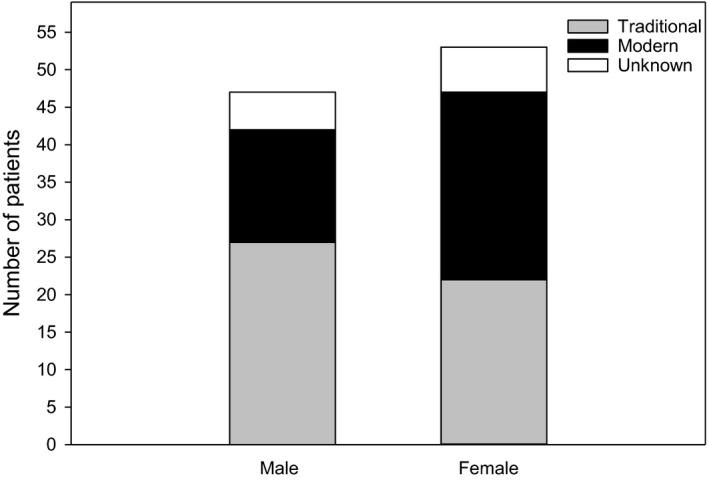
Sex of patients categorised by recruitment method.

There were 53 female (53%) and 47 male (47%) patients enrolled in the study (Figure 2). Eighty per cent more male patients were recruited by traditional compared with modern methods, which was in contrast with females where slightly fewer (12%) were recruited by traditional compared with modern methods. There were 67% more female compared with male patients recruited by modern methods, compared with 19% fewer female compared with male patients recruited by traditional methods (Figure 2).

**Figure 3:**
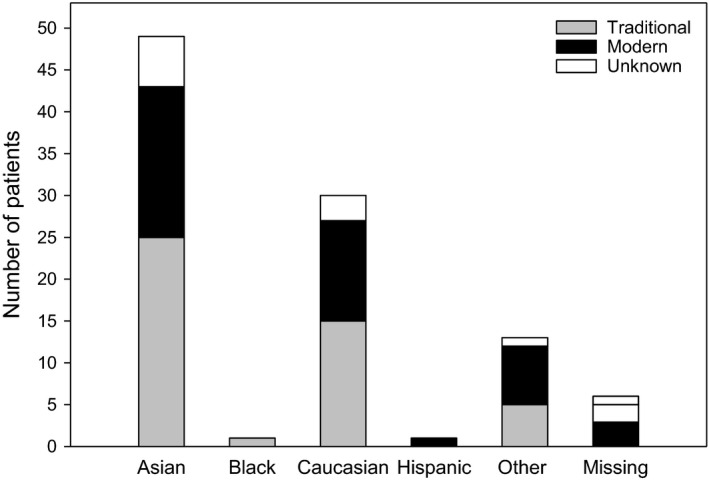
Patient race categorised by recruitment method.

**Figure 4:**
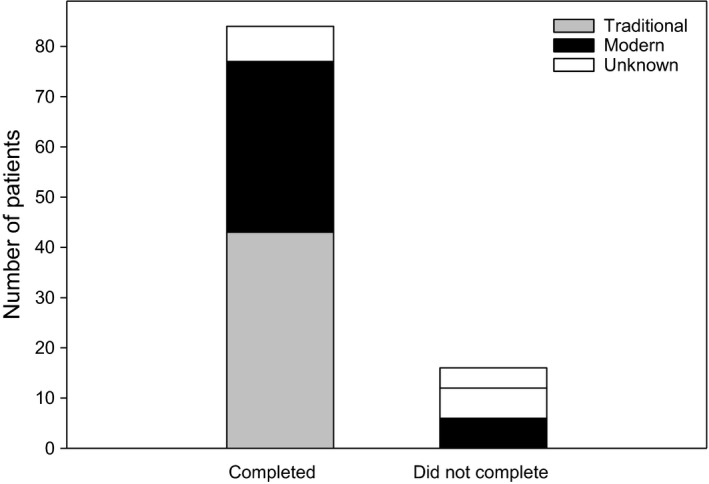
Number of patients who did and did not complete the study categorised by recruitment method.

The majority of patients were either Asian (49%) or Caucasian (30%), with the remaining being either Black (1%), Hispanic (1%), other (13%), or the data were missing (6%) (Figure 3). Similar proportions of each race were recruited by traditional and modern methods. Eighty three (83%) patients completed the study (Figure 4), which was not effected by recruitment method. Recruitment costs were approximately AUD$2,143 per enrolment for traditional methods and approximately AUD$2,494 per enrolment for modern methods. This equates to only $351 (16%) more per patient recruited by modern compared with traditional methods.

**Figure 5:**
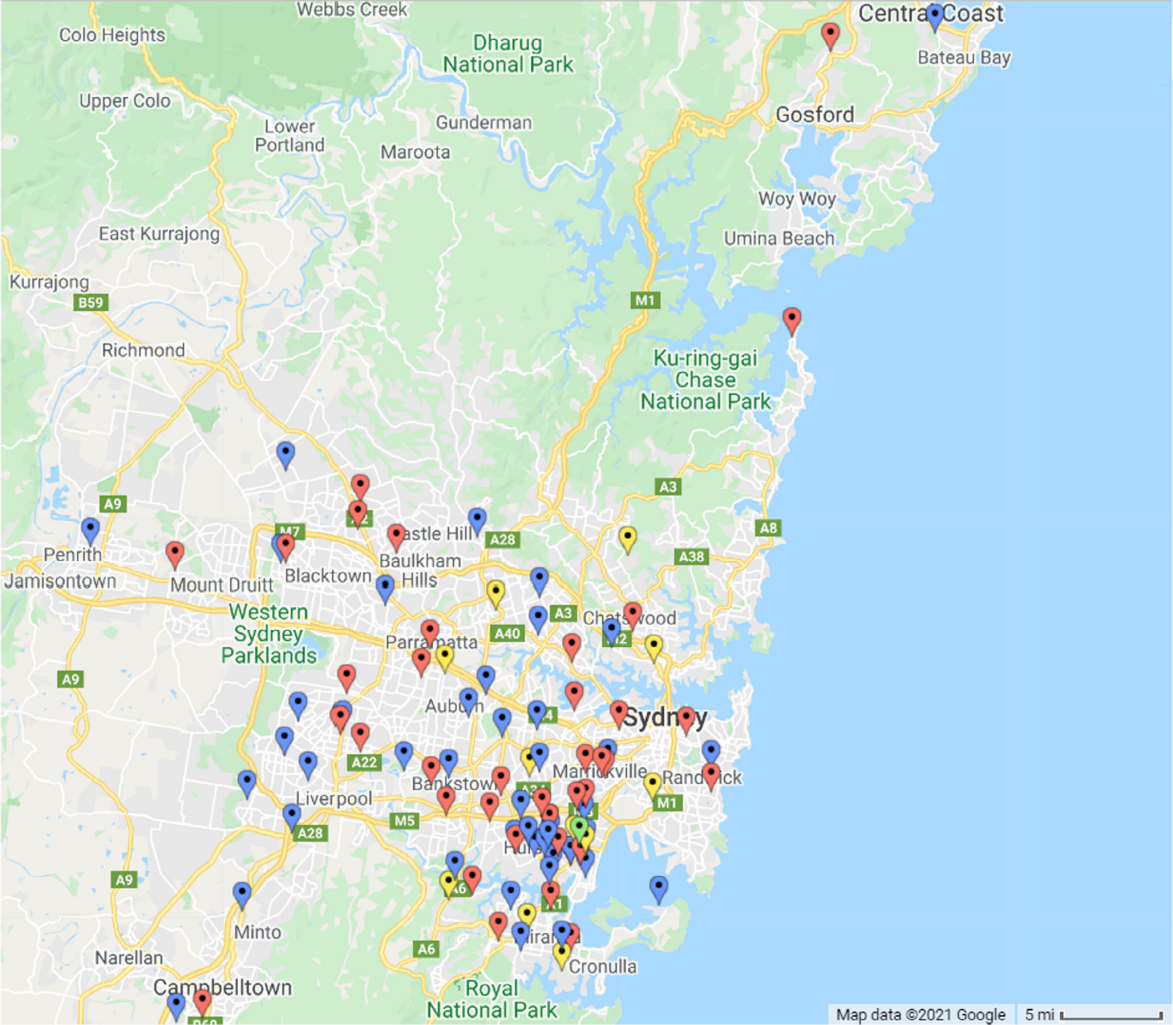
Map of greater Sydney, Australia and the surrounding area showing the distance patients travelled to participate in the study. The location of the trial site clinic is shown in green. Patient locations for those recruited by traditional methods are shown in blue, modern methods in red and unknown in yellow. Note that two patients who lived up to 100 km south of the clinic are not displayed on the map.

Figure 5 shows the distance patients travelled to take part in the study. Whilst the majority of patients travelled up to 30 km to the trial site clinic, several patients travelled much further, with two patients travelling approximately 100 km to be involved in the trial. The distance patients travelled was not effected by recruitment method.

## DISCUSSION

This is one of the first studies to compare the effectiveness of traditional versus modern recruitment methods to enrol patients with moderate eczema into a RCT. The recruitment of 100 adult patients required to reach statistical power was challenging, despite the fact that eczema is the most common inflammatory skin condition globally, affecting approximately 2–10% of adults.^22^ Many different advertising strategies, both traditional and modern, were required to meet the study target. This challenge is not unique and has been discussed for other diseases and conditions.^15^, ^16^, ^17^, ^18^, ^19^, ^20^

The majority of patients recruited for this RCT were under 40 years of age, which is consistent with the peak prevalence of eczema in adults which is from age 25–45 years, with it becoming less prevalent with increasing age.^23^ Interestingly, despite the rising popularity of social media and increased internet use especially amongst the younger generations,^12^ younger patients were similarly recruited by both traditional and modern methods, whilst older patients were predominately recruited by traditional methods. Almost twice as many men were recruited by traditional methods, whilst more women than men were recruited by modern methods. This can be attributed to the fact that older male patients were predominately recruited by traditional methods (10/13 of male Gen X and baby boomers).

The average age and race of the patient population reflected the demographics of the area surrounding the clinic in Kogarah, New South Wales, Australia.^24^ Most patients showed great motivation and compliance with the study requirements including twice daily applications of the study products and weekly visits to the clinic over the 5 week study duration (pre‐screening, washout and study period), with several patients travelling further than 30 km, and two patients willing to travel around 100 km to the clinic each way. RCTs provide an opportunity for patients to gain access to free specialist medical care involving either standard treatment for their condition or an investigational drug. In fact, emerging research suggests that patients participating in RCTs have better treatment outcomes than patients who have not participated.^25^

Whilst there were numerous expressions of interest for both recruitment methods, many patients did not meet the inclusion criteria as their eczema was not severe enough according to Eczema Area and Severity Index (EASI) scoring, or they did not have eczema at all. For traditional recruitment methods, radio advertisements and letterbox drops generated the most expressions of interest possibly due to the sheer number of letterbox flyer drops in the local area, and that radio advertising increased the reach of the study to the wider Sydney area compared to other advertising, which was predominately focussed around the clinic. Despite the high number of expressions of interest, radio advertisements and letterbox drops resulted in the lowest enrolment rates possibly due to the non‐targeted approach of these types of advertising. However, at the same time as the radio advertisements and letterbox drops, there were an increased number of word‐of‐mouth expressions of interest from people telling their family and friends about the study. Physician referrals resulted in the best enrolment rate for both recruitment methods due to the fact that patients were essentially ‘pre‐screened’ before being assessed for trial eligibility.

For modern recruitment methods, social media, including patient recruitment services, which also substantially relied on social media, generated many more expressions of interest than Google advertising. Websites and email generated relatively few expressions of interest and failed to enrol any patients. The only other recruitment study specific for dermatology found that a targeted advertising strategy on social media and search engines shortened the time to recruit patients with moderate to severe atopic dermatitis for a RCT.^26^

A limitation of the study is that the length of time taken for each advertising strategy to enrol patients was not measured, and therefore, it is unknown whether particular advertising strategies were quicker or more efficient at enrolling patients than others. Whilst this study focussed on Facebook and Instagram, since inception of the study many new platforms have been established for advertising, such as Snapchat and TikTok, which are widely used platforms that could be investigated. Twitter could also be useful.

## CONCLUSION

Despite the huge increase in the use of social media and the internet in recent times, our results suggest that traditional recruitment methods are certainly not obsolete or inferior, and that a balanced approach utilising both traditional and modern recruitment methods is required to enrol adult patients with moderate eczema into a RCT. Whilst these results may be unique to the nature of the patient population in this study, they suggest that investigators need to adapt to the ever changing landscape of social media and marketing, and be willing to embrace methods which may seem archaic.

## References

[1] Odgaard‐Jensen J , Vist GE , Timmer A *et al*. Randomisation to protect against selection bias in healthcare trials. Cochrane. Database. Syst. Rev. 2011;2011:MR000012. 10.1002/14651858.MR000012.pub3 PMC7150228 21491415

[2] Sorkness CA , Ford JG , Lemanske RF *et al*. Recruitment strategies in the asthma clinical research network. Control Clin Trials. 2001; 22: S222–35. 10.1016/s0197-2456(01)00172-6 11728626

[3] Spilker B , Cramer J . Patient recruitment in clinical trials. New York, NY, USA: Raven Press Ltd, 1992.

[4] McDonald AM , Knight RC , Campbell MK *et al*. What influences recruitment to randomised controlled trials? A review of trials funded by two UK funding agencies. Trials. 2006; 7: 9. 16603070 10.1186/1745-6215-7-9 PMC1475627

[5] Campbell MK , Snowdon C , Francis D *et al*. Recruitment to randomised trials: strategies for trial enrollment and participation study. The STEPS study. Health. Technol. Assess. 2007; 11: iii–126. 10.3310/hta11480 17999843

[6] Charlson ME , Horwitz RI . Applying results of randomised trials to clinical practice: impact of losses before randomisation. Br. Med. J. (Clin Res Ed). 1984; 289: 1281–4. 10.1136/bmj.289.6454.1281 PMC1443545 6437520

[7] Carlisle B , Kimmelman J , Ramsay T *et al*. Unsuccessful trial accrual and human subjects protections: an empirical analysis of recently closed trials. Clin. Trials. 2015; 12: 77–83. 25475878 10.1177/1740774514558307 PMC4516407

[8] Altman DG . Statistics and ethics in medical research III. How large a sample? Br. Med. J. 1980; 281: 1336–8. 7437789 10.1136/bmj.281.6251.1336 PMC1714734

[9] Treweek S , Lockhart P , Pitkethly M *et al*. Methods to improve recruitment to randomised controlled trials: Cochrane systematic review and meta‐analysis. BMJ Open. 2013; 3: e002360. 10.1136/bmjopen-2012-002360 PMC3586125 23396504

[10] Isaksson E , Wester P , Laska AC *et al*. Identifying important barriers to recruitment of patients in randomised clinical studies using a questionnaire for study personnel. Trials. 2019; 20: 618. 31666093 10.1186/s13063-019-3737-1 PMC6822437

[11] Watson JM , Torgerson DJ . Increasing recruitment to randomised trials: a review of randomised controlled trials. BMC. Med. Res. Methodol. 2006; 6: 34. 16854229 10.1186/1471-2288-6-34 PMC1559709

[12] Arigo D , Pagoto S , Carter‐Harris L *et al*. Using social media for health research: Methodological and ethical considerations for recruitment and intervention delivery. Digit. Health. 2018; 4: 2055207618771757. 29942634 10.1177/2055207618771757 PMC6016568

[13] Caplan A , Friesen P . Health disparities and clinical trial recruitment: Is there a duty to tweet? PLoS. Biol. 2017; 15: e2002040. 28249024 10.1371/journal.pbio.2002040 PMC5331960

[14] Lohse B . Facebook is an effective strategy to recruit low‐income women to online nutrition education. J. Nutr. Educ. Behav. 2013; 45: 69–76. 23305805 10.1016/j.jneb.2012.06.006

[15] Guillory J , Wiant KF , Farrelly M *et al*. Recruiting hard‐to‐reach populations for survey research: Using Facebook and Instagram advertisements and in‐person intercept in LGBT bars and nightclubs to recruit LGBT young adults. J. Med. Internet. Res. 2018; 20: e197. 29914861 10.2196/jmir.9461 PMC6028767

[16] Admon L , Haefner JK , Kolenic GE *et al*. Recruiting pregnant patients for survey research: A head to head comparison of social media‐based versus clinic‐based approaches. J. Med. Internet. Res. 2016; 18: e326. 28003174 10.2196/jmir.6593 PMC5215244

[17] Darmawan I , Bakker C , Brockman TA *et al*. The role of social media in enhancing clinical trial recruitment: Scoping review. J. Med. Internet. Res. 2020; 22: e22810. 33104015 10.2196/22810 PMC7652693

[18] Frandsen M , Thow M , Ferguson SG . The effectiveness of social media (Facebook) compared with more traditional advertising methods for recruiting eligible participants to health research studies: A randomized, controlled clinical trial. JMIR. Res. Protoc. 2016; 5: e161. 27511829 10.2196/resprot.5747 PMC4997003

[19] Moreno MA , Waite A , Pumper M *et al*. Recruiting adolescent research participants: In‐person compared to social media approaches. Cyberpsychol. Behav. Soc. Netw. 2017; 20: 64–7. 27976951 10.1089/cyber.2016.0319

[20] Topolovec‐Vranic J , Natarajan K . The use of social media in recruitment for medical research studies: a scoping review. J. Med. Internet. Res. 2016; 18: e286. 27821383 10.2196/jmir.5698 PMC5118584

[21] Spada F , Harrison IP , Barnes TM *et al*. A daily regimen of a ceramide‐dominant moisturizing cream and cleanser restores the skin permeability barrier in adults with moderate eczema: a randomized trial. Dermatol. Ther. 2021; 34: e14970. 33984185 10.1111/dth.14970 PMC8459234

[22] Barbarot S , Auziere S , Gadkari A *et al*. Epidemiology of atopic dermatitis in adults: results from an international survey. Allergy 2018; 73: 1284–93. 29319189 10.1111/all.13401

[23] Ali F , Vyas J , Finlay AY . Counting the burden: atopic dermatitis and health‐related quality of life. Acta. Derm. Venereol. 2020; 100: adv00161. 32412644 10.2340/00015555-3511 PMC9189752

[24] https://quickstats.censusdata.abs.gov.au/census_services/getproduct/census/2016/quickstat/SED10040. Accessed on 18 May, 2021.

[25] Nijjar SK , D’Amico MI , Wimalaweera NA *et al*. Participation in clinical trials improves outcomes in women’s health: A systematic review and meta‐analysis. BJOG 2017; 124: 863–71. 28194870 10.1111/1471-0528.14528

[26] Katz BE , Eiken A , Misev V *et al*. Optimize clinical trial recruitment with digital platforms. Dermatol. Times. 2019;40:January.

